# Investigating the impact of early adversity on perceived support from parents and friends in preadolescence: Do genetic predispositions and timing of exposure matter?

**DOI:** 10.1002/jcv2.70090

**Published:** 2026-01-20

**Authors:** Christina Y. Cantave, Marthe de Roo, Charlotte Vrijen, Tina Kretschmer

**Affiliations:** ^1^ Institute of Child Development University of Minnesota Twin‐Cities Minneapolis Minnesota USA; ^2^ Department of Pedagogy and Educational Sciences University of Groningen Groningen Netherlands; ^3^ Department of Developmental Psychology University of Groningen Groningen Netherlands

**Keywords:** adverse childhood experiences (ACEs), gene‐environment interplay, polygenic scores, social support, sources of support

## Abstract

**Background:**

Perceived social support contributes to youth resilience, but perceptions of support differ substantially between youth. Genetic predispositions and early life adversity are potential sources for this variation, but research has yet to test gene‐environment interplay comprehensively. We examined how early adversity and genetic predispositions for educational attainment, internalizing, and externalizing behaviours uniquely and jointly predict parents and friends support at age 11 and explored associations for different timing of adversity.

**Methods:**

Participants were drawn from TRAILS, a longitudinal study of Dutch adolescents. Adversity was measured during the perinatal period (pregnancy to 1‐month postdelivery) and childhood (ages 0–11) through Preventive Child Healthcare records and parental interviews at age 11. Participants' genotype data were used to derive polygenic scores. Perceived social support from mothers (*n* = 1634), fathers (*n* = 1609) and friends (*n* = 1634) were self‐reported at age 11.

**Results:**

Genetic predispositions for internalizing and externalizing problems were associated with higher childhood adversity and were indirectly related to lower perceived social support. Exposure to perinatal adversity was related to higher maternal support during adolescence, while childhood adversity was associated with less paternal support. These associations further varied based on specific subtypes of adversity, but not according to adolescents' genetic predispositions.

**Conclusion:**

Adolescents' genetic liabilities to internalizing and externalizing behaviours predicted early adversity, which correlated with parental but not friends' support. These associations were found to vary based on the timing and nature of adversity, underscoring the need for future studies to more consistently account for these factors and to examine their underlying mechanisms.

## Key points

**What's known?**

- Youth exposed to adverse experiences are more likely to perceive lower social support.

**What's new?**

- Youth with greater genetic predispositions for internalizing and externalizing problems were more likely to experience psychosocial adversity and, indirectly, to perceive lower social support, emphasizing the need to mitigate these risks.
- The associations between genetic predispositions and adversity varied by the timing and subtypes of adversity, highlighting the importance of considering these factors in future studies.
- Exposure to prenatal adversity was unexpectedly associated with greater perceived maternal support in preadolescence.

**What's relevant?**

- Low family income, parental divorce, and parental history of externalizing behaviours were linked to lower perceived paternal support, underscoring the need to strengthen father‐child bonds and improve youths' perceptions of support.

## Graphical abstract

This study found that youth with a higher genetic risk for internalizing and externalizing problems were more likely to face adversity within their home, highlighting the need for risk mitigation. These associations varied by adversity type and timing. Additionally, low family income, parental divorce, and externalizing behaviours were linked to lower perceived paternal support, emphasizing the need for targeted interventions to strengthen father‐child relationships and improve perceived social support.

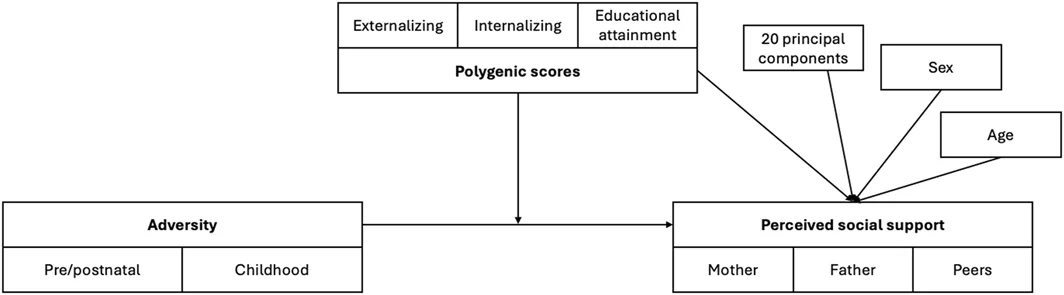

## INTRODUCTION

Social support is widely recognized as a critical resource that promotes developmental competence, healthy development and resilience in children and adolescents (Gayman et al., 2011; Rueger et al., 2016). Perceived social support, defined as feeling loved, cared for, valued, and connected to a mutually supportive social network (Taylor, 2011), primarily comes from parents in early life, with friends becoming an additional source of support during adolescence (Levitt et al., 1993). Adolescents who feel supported by their parents and friends tend to report better mental health and higher well‐being in both adolescence and young adulthood (Jakobsen et al., 2022; Rueger et al., 2016), even in the aftermath of adverse childhood experiences (ACEs; Forster et al., 2020; Ragavan et al., 2020). Yet, despite its theorized stress‐buffering role (Cohen & Wills, 1985), evidence for the protective role of perceived social support remains mixed (Cantave et al., 2024; Rueger et al., 2016). These inconsistent results suggest that perceived social support does not operate uniformly across individuals, highlighting the need to better understand individual differences in youth perceptions of social support. Identifying the sources of these variations is important, as it can inform future mechanistic studies investigating the protective role of perceived social support and ultimately guide targeted interventions to promote youth resilience.

Early life experiences are thought to lay the foundation for how individuals perceive the availability of support in their social networks (Taylor, 2011). It has been proposed that exposure to ACEs in the home can foster an insecure attachment style, leading to negative self and other beliefs that impair cognitive processes and result in adolescents' lower perceived support (Blain et al., 1993; Kitamura et al., 2002; Lakey & Cassady, 1991). Alternatively, ACEs may induce maladaptive neurocognitive changes, impacting social and behavioural functioning in ways that limit adolescents' social networks and support (McCrory et al., 2022). Consistent with these hypotheses, McCoy et al. (2020) found that adolescents exposed to more ACEs perceived less support from their family, school and community. Similarly, Turner et al. (2017) documented that adolescents who experienced either an increase in ACEs or consistently high ACEs over 2 years reported decreasing social support from families and friends, unlike their counterparts with continually low ACEs exposure. However, contrasting results also emerged, with one study reporting that ACEs was concurrently associated with higher peer support among disadvantaged adolescents (Yearwood et al., 2019), and others reporting nonsignificant associations (Zerach & Elklit, 2020; Zinn et al., 2017). One factor that may help explain these mixed findings is the timing of exposure to ACEs. For instance, in a recent study, exposure to lower family socioeconomic status during mid‐adolescence, but not early childhood, correlated with lower perceived social support both concurrently and from mid‐to‐late adolescence (Cantave et al., 2024), suggesting that perceptions of social support might be more sensitive to recent than to earlier exposure to ACEs. It is unclear whether these results extend to *preadolescence* and whether associations might differ by source of support (i.e., mother, father, and friends) or by timing of exposure to ACEs (perinatal vs. childhood). Furthermore, another confounder that has been largely overlooked in these previous studies is the partial genetic basis of variations in perceived social support.

Twin studies indicate that genetic factors contribute to 40%–55% of variation in perceived social support during adolescence (Matthews et al., 2016; Wang et al., 2017). These studies also show that mental health outcomes, particularly depressive symptoms and well‐being, and perceived social support share overlapping genetic influences (Wang et al., 2017). This genetic correlation suggests that some of the same genetic factors that predispose individuals to poor mental health may also contribute to lower perceptions of social support. While these findings provide compelling evidence for a shared genetic architecture, the specific genetic variants involved remain largely unknown. Genome‐wide association studies (GWAS), especially those based on large samples (i.e., GWAS on educational attainment, internalizing and externalizing behaviours), offer a promising avenue to identify such markers via the estimation of polygenic scores. Polygenic scores, which index an individual's cumulative genetic propensity for various traits (Domingue et al., 2015), allow to investigate how genetic predispositions may affect social support. For instance, higher polygenic scores for educational attainment may correlate with higher perceived support from families and friends, as these predispositions are related to better cognitive abilities, self‐control, and interpersonal skills (Belsky et al., 2016; Cohen et al., 1986; Domingue et al., 2015; La Fleur & Salthouse, 2017; Ronen et al., 2016). In contrast, higher genetic predispositions for internalizing and externalizing behaviours may negatively impact adolescents' perception of social support, potentially even before these behaviours fully manifest. Such genetic predispositions may be linked to impaired cognitive processes (e.g., attention and memory) and personal characteristics (e.g., neuroticism, hostility, low extraversion) that may contribute to less perceived support (Karlsson Linnér et al., 2021; Lakey, 2013; Lakey & Cassady, 1991; Tubbs & Sham, 2023). Despite this, direct investigations linking polygenic indices to adolescents' perceived social support are lacking. Yet, such investigation could significantly advance our understanding of the genetic underpinnings of social support during adolescence.

**FIGURE 1:**
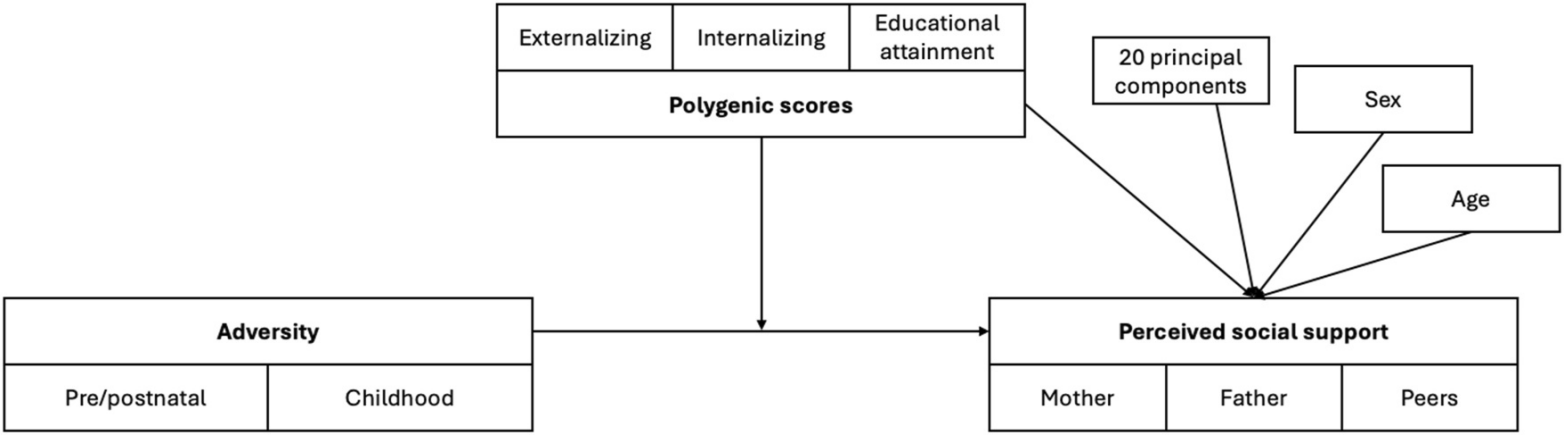
Predicted associations between the study main variables.

Although previous studies have investigated the contribution of ACEs and genetic factors in isolation, these factors likely correlate and interact in ways that account for individual differences in perceived social support. By identifying individuals who are more genetically susceptible to the negative outcomes of ACEs, gene‐environment interaction models can help clarify for whom ACEs are more likely to erode perceived social support and for whom they are less impactful. Such fundamental work can, in turn, inform future mechanistic studies investigating the protective role of perceived social support following ACEs. Gene‐environment interactions may manifest in various forms. They could take the form of a *compensation effect* whereby genetically advantaged adolescents (i.e., higher polygenic score for educational attainment and lower polygenic score for externalizing and internalizing behaviours) are buffered against the negative effect of ACEs on their perceived support from parents and friends. Contrastingly, reflecting a *suppression effect*, the beneficial effect of higher genetic predispositions for educational attainment and lower genetic predispositions for externalizing and internalizing behaviours on adolescents' perceived social support may be reduced in more adverse environments in comparison to more neutral contexts. Despite their relevance for understanding the origins of individual differences in youth resilience, these hypotheses have rarely been examined before. To address this gap, the current study investigated how ACEs and the polygenic scores for educational attainment, internalizing, and externalizing behaviours uniquely and jointly predict adolescents' reports of support from their parents and friends at age 11 (see Figure 1). Additionally, we assessed these associations for different periods of exposure to ACEs (i.e., the perinatal period vs. the childhood period [0–11 years old]). Given prior evidence linking genetic predispositions to ACEs exposure (Baldwin et al., 2022; Zwicker et al., 2020), we tested and adjusted for gene‐environment correlations to avoid biases (Brendgen et al., 2012). The study focussed on perceived social support at age 11, as this was the only wave with data on both parental and friends' support.

## MATERIALS AND METHODS

### Participants

Participants were drawn from the TRacking Adolescents' Individual Lives Survey (TRAILS), a prospective cohort study of Dutch adolescents, with bi‐ or triennial follow‐up assessments. TRAILS consists of a population and a high‐risk cohort: the population cohort comes from five municipalities in the north of the Netherlands, including urban and rural areas. Initially, 135 primary schools were approached of which 122 agreed to participate. In brief, a total of 2935 children were invited to participate of whom 2229 (51% female) did so at T1 (for more details on the selection procedure, see Huisman et al., 2008). Data collection at the first assessment wave (T1) took place in 2001 and 2002 (mean age = 11.11; SD = 0.55; range = 10.01–12.58). The TRAILS population cohort was complemented by a high‐risk cohort selected based on contact with child and adolescent mental health services before age 11. This high‐risk cohort was set up in 2004, with the inclusion of 543 children (response rate 43%). Boys were overrepresented (66%), in line with sex ratios for the most common childhood psychopathologies. About 16% of children from both cohorts were from low‐income households (< €1,135, which approximately amounts to a welfare payment). Comparable to the population sample, follow‐up data collection waves for the high‐risk cohort occurred at intervals of 2–3 years but lag by approximately one assessment wave. In both cohorts, similar measures were used to collect data and participants underwent assessments at roughly the same ages. Ethics approval for TRAILS was obtained from the Dutch national ethics committee CCMO and both parents and children provided informed consent. The present study used data from the first assessment (across both cohorts), in addition to genetic data collected from participants with European ancestry at T3 (*n* = 1353 from the population cohort and *n* = 341 from the high‐risk cohort). Details about the study and attrition are published elsewhere (Oldehinkel et al., 2015). Full information maximum likelihood was used to estimate our models, yielding analytical sample sizes of *n* = 1634 for analyses involving perceived social support from mothers and friends, and *n* = 1609 for those involving perceived social support from fathers.

### Measures

#### Prenatal and childhood adversity

Prenatal adversity was derived using retrospective data collected through home interviews with parents (primarily the mothers, 95%) at age 11 (T1) by well‐trained interviewers. These indicators included mother's hospitalization during pregnancy (16%), children's hospitalization for 10 days or more immediately after birth (8%), maternal physical and psychological problems during the first month after delivery (22.4%). These items were dummy coded (0 = little to no exposure, 1 = exposure). In addition, birth weight and gestational age, evaluated by the obstetrician or midwife, were extracted from Preventive Child Healthcare (PCH) files (Reijneveld et al., 2004). In cases of missing PCH data (*n* = 82), imputation was performed using birth weight and gestational age data obtained from parents, which highly correlated (*r* > 0.87) with the PCH data. From this information, two dummy coded indicators were derived—preterm birth (≤ 33 weeks; 2%) and low birth weight (≤ 2500 g [≈5.51 lb]; 12%).

Childhood adversity was also derived from information obtained during the parental interview conducted at age 11. The score was based on retrospective reports of adverse family events that occurred when the child was aged between 0 and 11. These events included parental divorce (22%), out‐of‐home placement (2%), parental history of mental health problems (externalizing (12%) and internalizing problems (40%)), and low family income (< €1135 per month at T1; 14%; see Appendix S1 for more details). As with the perinatal adversity score, all items were dummy coded (0 = little to no exposure, 1 = exposure).

Consistent with best practice (Wright & Schwartz, 2021), all items were included in confirmatory factor analyses to derive robust and cohesive latent factors of adversity during the perinatal and childhood periods (see Appendix S1 for more details). Good model fit and parsimony are generally indicated by a nonsignificant chi‐square statistic (*χ* ^2^), a comparative fit index (CFI) and Tucker Lewis index (TLI) ≥ 0.90, a root mean square error of approximation (RMSEA) < 0.06 and a standardized root mean square residual (SRMR) < 0.08 (Hu & Bentler, 1999). Adequate model fit was observed for both perinatal [*χ* ^2^ (5) = 46.12, *p* = 0.001; RMSEA = 0.06; CFI = 0.97; TLI = 0.94; SRMR = 0.06] and childhood [*χ* ^2^ (5) = 54.18, *p* = 0.001; RMSEA = 0.06; CFI = 0.96; TLI = 0.91; SRMR = 0.05] adversity factors. The estimated factors for perinatal (Mean [standard deviation; SD] = 0.12 [0.57]) and childhood adversity (Mean [SD] = 0.06 [0.37]) were saved for use in the subsequent analyses described below.

#### Polygenic scores

Genotyping procedures and polygenic scores validation are described in detail in the Appendices S2 and S3 and Table S1. The polygenic scores were estimated based on summary statistics from available genome‐wide association studies (GWAS) of internalizing problems (Howard et al., 2019), externalizing problems (Karlsson Linnér et al., 2021) and educational attainment (Okbay et al., 2022). The externalizing GWAS included TRAILS data, and MetaSubtract v1.60 (Nolte, 2020) was used to subtract TRAILS data from the summary statistics. We used LDPred2‐auto to calculate the three polygenic scores, which represent weighted sums of alleles based on effect sizes from each GWAS's summary statistics and a linkage disequilibrium matrix. LDpred2‐auto automatically estimates SNP‐heritability (*h* ^*2*^) and the proportion of causal variants (*p*) from the data, eliminating the need for a validation dataset to tune these parameters (Privé et al., 2021). As done previously (de Roo et al., 2023), only HapMap3+ variants that had undergone rigorous quality control were included in the polygenic scores estimation, as they provide good coverage of the whole genome. We used the LD reference panel, sourced from European individuals within the UK Biobank, as supplied by the developers of LDPRED 2. We used the polygenic score with all available variants included, that is, did not apply a p‐threshold for inclusion.

#### Perceived social support

Social support was reported by adolescents at age 11 using an adapted version of the Social Production Functions questionnaire (Ormel et al., 1997). This questionnaire included four items assessing adolescents' perceived social support (e.g., *my [mother] enjoys being with me*; *my [mother] takes my feelings into account*; *I can really trust my [mother]*; *my [mother] helps me when something is wrong*) from their mothers, fathers and friends, respectively. Items were rated on a 5‐point Likert‐type scale ranging from 1 (*Never*) to 5 (*always*) and were averaged to create a total score for the mother (Mean [SD] = 4.36 [0.64]; ∝ = 0.78), father (Mean [SD] = 4.19 [0.80]; ∝ = 0.84) and friends (Mean [SD] = 3.91 [0.79]; ∝ = 0.84), respectively.

### Statistical analyses

Preliminary analyses were first conducted to assess potential associations between the main variables and covariates (i.e., sex and age measured at T1). Therefore, the identified covariates, in addition to 20 principal components to account for population stratification were controlled for in the subsequent analyses. Next, we tested the effects of adversity and the polygenic scores on perceived social support using linear regression analysis in Mplus version 8.1.7. Specifically, the independent and interactive effects of the predictors were added sequentially to the models. Given the skewed distribution of the adversity factors and the social support scales, maximum likelihood with robust standard errors was used as an estimator in our models.

## RESULTS

### Associations between the main variables and covariates

Our preliminary analyses revealed that adolescent boys were more likely to report lower support from their parents and friends than girls (see Table 1). We also found that age was related to greater exposure to childhood adversity and lower perceived support from mothers. Thus, sex and age were adjusted for in the regression models. As shown in Table 1, a modest association emerged between perinatal adversity and childhood adversity reported by parents, suggesting some degree of continuity as children who experienced higher adverse experiences during the perinatal period were more likely to face adversity in later childhood (ages 0–11). Internalizing and externalizing polygenic scores were associated with childhood adversity, indicating that children with higher genetic predispositions for these problems were more likely to face adversity during childhood. These associations were not found for the educational attainment PGS or perinatal adversity. Moreover, higher perinatal adversity was related to greater perceived maternal support at age 11, while greater childhood adversity correlated with lower perceived paternal support at the same age. Perinatal and childhood adversity did not co‐vary with support from friends during preadolescence. Moderate‐to‐strong correlations were observed between perceived support from different providers.

**TABLE 1:** Associations between the main variables and the covariates.

	1	2	3	4	5	6	7	8	9	10
Sex (0 = female)	—	0.03†	−0.01	−0.02	−0.04	0.01	−0.02	−0.09***	−0.04*	−0.19***
Age		—	0.02	0.07***	0.04 †	−0.01	0.02	−0.05**	−0.02	−0.01
Perinatal adversity			—	0.07***	0.03	0.02	0.01	0.04*	0.01	0.02
Childhood adversity (0–11 years old)				—	0.13***	0.17***	−0.04†	0.003	−0.07***	0.01
Internalizing polygenic score					—	0.25***	−0.01	0.004	0.01	−0.02
Externalizing polygenic score						—	0.01	−0.01	−0.03	0.02
Educational attainment polygenic score							—	0.02	−0.01	−0.01
PS from mothers								—	0.59***	0.37***
PS from fathers									—	0.37***
PS from friends										—

*Note*: Standardized beta coefficients; ***p ≤ 0.001, **p ≤ 0.01, *p ≤ 0.05, † *p* ≤ 0.10. Significant results are indicated in boldface.

Abbreviation: PS, perceived support.

### How do genetic predisposition, perinatal adversity, and childhood adversity independently and jointly relate to adolescents' perceived social support?

We first tested the additive effect of perinatal and childhood adversity by incorporating both in model 1 (see Table 2) as predictors of adolescents' perceived support from mothers, fathers, and friends. The positive association between perinatal adversity and support from mothers remained significant, after adjustments for sex, age, and childhood adversity. Moreover, a unique but negative association was observed between childhood adversity and support from fathers. However, neither perinatal nor childhood adversity were associated with support from friends. Subsequently, we introduced the internalizing polygenic score in model 2 to examine its independent effect on support as well as the interaction between genetic and environmental risk in model 3. The internalizing polygenic score did not predict support from parents or friends, nor were the interaction terms between adversity factors and the polygenic score significant, indicating that the polygenic score did not modulate the observed associations between ACEs and adolescents perceived parental support.

**TABLE 2:** Main and interactive effects of adversity and the internalizing polygenic score on perceived support from parents and friends.

	Model 1		Model 2		Model 3	
	*β*	*P*	*β*	*p*	*β*	*p*
PS mothers (*N* = 1634)						
Sex	−0.095	0.001	−0.095	0.001	−0.096	0.000
Age	−0.089	0.001	−0.089	0.001	−0.089	0.000
Perinatal adversity	**0.061**	**0.010**	**0.061**	**0.013**	**0.061**	**0.010**
Childhood adversity	−0.011	0.703	−0.010	0.676	−0.014	0.625
INT polygenic score			−0.003	0.906	−0.003	0.907
Perinatal adversity × INT polygenic score					0.001	0.978
Childhood adversity × INT polygenic score					0.021	0.466
	*R* ^2^ = 0.028		*R* ^2^ = 0.028		*R* ^2^ = 0.029	
PS fathers (*N* = 1609)						
Sex	−0.049	0.046	−0.049	0.047	−0.049	0.048
Age	−0.032	0.213	−0.032	0.207	−0.032	0.212
Perinatal adversity	0.043	0.086	0.043	0.086	0.043	0.083
Childhood adversity	**−** **0.061**	**0.032**	**−** **0.062**	**0.029**	**−** **0.061**	**0.033**
INT polygenic score			0.011	0.667	0.011	0.663
Perinatal adversity × INT polygenic score					−0.024	0.433
Childhood adversity × INT polygenic score					−0.006	0.840
	*R* ^2^ = 0.015		*R* ^2^ = 0.016		*R* ^2^ = 0.016	
PS friends (*N* = 1634)						
Sex	−0.175	0.001	−0.176	0.001	−0.177	0.001
Age	−0.026	0.284	−0.025	0.304	−0.026	0.293
Perinatal adversity	0.035	0.148	0.035	0.140	0.035	0.145
Childhood adversity	−0.028	0.306	−0.024	0.378	−0.028	0.300
INT polygenic score			−0.034	0.160	−0.035	0.155
Perinatal adversity × INT polygenic score					−0.029	0.236
Childhood adversity × INT polygenic score					0.027	0.298
	*R* ^2^ = 0.046		*R* ^2^ = 0.047		*R* ^2^ = 0.049	

*Note*: All models are adjusted for sex, age and 20 principal components. Significant results are indicated in boldface.

Abbreviations: INT polygenic score, polygenic score for internalizing problems; PS, perceived support; β, standardized beta coefficients.

Comparable analyses were conducted for the externalizing (see Table 3, models 2 and 3) and the educational attainment polygenic scores (see Table 4, models 2 and 3), which yielded no significant associations with support from parents or friends, whether considered independently or in conjunction with perinatal or childhood adversity.

**TABLE 3:** Main and interactive effects of adversity and the externalizing polygenic score on perceived support from parents and friends.

	Model 1		Model 2		Model 3	
	*β*	*P*	*β*	*p*	*β*	*p*
PS mothers (*N* = 1634)						
Sex	−0.095	0.001	−0.095	0.001	−0.095	0.001
Age	−0.089	0.001	−0.089	0.001	−0.089	0.001
Perinatal adversity	**0.061**	**0.010**	**0.061**	**0.010**	**0.061**	**0.009**
Childhood adversity	−0.011	0.703	−0.010	0.728	−0.011	0.698
EXT polygenic score			−0.006	0.814	−0.006	0.804
Perinatal adversity × EXT polygenic score					−0.009	0.683
Childhood adversity × EXT polygenic score					0.007	0.798
	*R* ^2^ = 0.028		*R* ^2^ = 0.028		*R* ^2^ = 0.028	
PS fathers (*N* = 1609)						
Sex	−0.049	0.046	−0.049	0.048	−0.049	0.048
Age	−0.032	0.213	−0.032	0.205	−0.032	0.210
Perinatal adversity	0.043	0.086	0.043	0.083	0.044	0.078
Childhood adversity	**−** **0.061**	**0.032**	**−** **0.057**	**0.043**	**−** **0.056**	**0.050**
EXT polygenic score			−0.019	0.443	−0.020	0.427
Perinatal adversity × EXT polygenic score					−0.017	0.488
Childhood adversity × EXT polygenic score					−0.005	0.869
	*R* ^2^ = 0.015		*R* ^2^ = 0.016		*R* ^2^ = 0.016	
PS friends (*N* = 1634)						
Sex	−0.175	0.001	−0.176	0.001	−0.176	0.001
Age	−0.026	0.284	−0.026	0.297	−0.026	0.297
Perinatal adversity	0.035	0.148	0.034	0.151	−0.034	0.150
Childhood adversity	−0.028	0.306	−0.031	0.261	−0.037	0.192
EXT polygenic score			0.020	0.415	−0.020	0.416
Perinatal adversity × EXT polygenic score					−0.005	0.836
Childhood adversity × EXT polygenic score					0.033	0.240
	*R* ^2^ = 0.046		*R* ^2^ = 0.046		*R* ^2^ = 0.047	

*Note*: All models are adjusted for sex, age and 20 principal components. Significant results are indicated in boldface.

Abbreviations: EXT polygenic score, polygenic score for externalizing problems; PS, perceived support; β = standardized beta coefficients.

**TABLE 4:** Main and interactive effects of adversity and the educational attainment polygenic score on perceived support from parents and friends.

	Model 1		Model 2		Model 3	
	β	*P*	β	*p*	β	*p*
PS mothers (*N* = 1634)						
Sex	−0.095	0.001	−0.095	0.001	−0.095	0.001
Age	−0.089	0.001	−0.090	0.001	−0.090	0.001
Perinatal adversity	**0.061**	**0.010**	**0.061**	**0.010**	**0.062**	**0.010**
Childhood adversity	−0.011	0.703	−0.010	0.733	−0.010	0.722
EA polygenic score			0.020	0.389	0.019	0.407
Perinatal adversity × EA polygenic score					−0.017	0.452
Childhood adversity × EA polygenic score					−0.009	0.708
	*R* ^2^ = 0.028		*R* ^2^ = 0.029		*R* ^2^ = 0.029	
PS fathers (*N* = 1609)						
Sex	−0.049	0.046	−0.049	0.045	−0.049	0.048
Age	−0.032	0.213	−0.031	0.217	−0.031	0.218
Perinatal adversity	0.043	0.086	0.043	0.085	0.044	0.085
Childhood adversity	**−0.061**	**0.032**	**−0.061**	**0.031**	**−0.062**	**0.029**
EA polygenic score			−0.014	0.578	−0.015	0.548
Perinatal adversity × EA polygenic score					−0.010	0.694
Childhood adversity × EA polygenic score					−0.028	0.349
	*R* ^2^ = 0.015		*R* ^2^ = 0.016		*R* ^2^ = 0.017	
PS friends (*N* = 1634)						
Sex	−0.175	0.001	−0.176	0.001	−0.177	0.001
Age	−0.026	0.284	−0.026	0.295	−0.026	0.289
Perinatal adversity	0.035	0.148	0.035	0.144	−0.036	0.139
Childhood adversity	−0.028	0.306	−0.029	0.283	−0.028	0.296
EA polygenic score			−0.025	0.332	−0.024	0.354
Perinatal adversity × EA polygenic score					−0.022	0.386
Childhood adversity × EA polygenic score					0.019	0.510
	*R* ^2^ = 0.046		*R* ^2^ = 0.047		*R* ^2^ = 0.047	

*Note*: All models are adjusted for sex, age and 20 principal components. Significant results are indicated in boldface.

Abbreviations: EA polygenic score, polygenic score for educational attainment; PS, perceived support; β, standardized beta coefficients.

### Specificity analyses

To verify the robustness of the significant correlations, we conducted additional post hoc analyses. First, we tested whether the correlations between adversity, genetic predispositions and support were consistent across specific subtypes of adversity measured during the perinatal period and childhood. As shown in Table S2, a significant association was noted between the internalizing polygenic score and maternal physical or psychological problems during the first month post‐delivery. Specifically, children with higher genetic propensities for internalizing problems were more frequently subjected to this adverse experience during the postnatal period. Additionally, greater genetic propensity for higher educational attainment was linked to an increased likelihood of preterm birth. Preterm birth also correlated with increased perceived support from mothers at age 11, whereas experiencing maternal physical or psychological problems during the first month after delivery was associated with greater perceived support from both parents.

Table S3 shows that genetic predispositions for internalizing and externalizing problems were significantly associated with greater exposure to all childhood adversity subtypes, with the exception of out‐of‐home placement. On the other hand, genetic predispositions for higher educational attainment were specifically linked to reduced chances of exposure to parental history of externalizing problems. Additionally, parental history of internalizing problems was related to lower perceived support from mothers. By contrast, low family income, parental divorce, and parental history of externalizing problems were associated with decreased perceived support from fathers.

Secondly, we explored whether the PGSs were indirectly associated with perceived social support through their effects on their respective phenotypes assessed at age 11. Higher genetic predispositions for internalizing and externalizing problems were indirectly associated to lower perceived support from parents and friends via their effects on internalizing and externalizing behaviours. No significant indirect effect emerged for the educational attainment PGS (see Table S4 in the SI for more information).

## DISCUSSION

Anchored in a resilience approach, the current study examined how ACEs and genetic predispositions for educational attainment, internalizing and externalizing behaviours were independently and jointly associated with perceived support from parents and friends at age 11. In addition, we explored whether these associations varied based on the timing of ACEs. Our findings indicated that higher genetic predispositions for internalizing and externalizing behaviours were associated with greater exposure to childhood adversity and were indirectly related to lower perceived social support. Conversely, exposure to perinatal adversity was related to greater perceived support from mothers during preadolescence, while experiencing greater childhood adversity was associated with less support from fathers. Specificity analyses further indicated that these patterns of association varied depending on the subtype of adversity experienced. Contrary to expectations, the observed associations between perinatal and childhood ACEs with perceived parental support were not found to be modulated by genetic predispositions.

We focus our discussion on three noteworthy findings. First, the current study found no significant direct associations between the polygenic scores for educational attainment, internalizing, and externalizing behaviours and perceived social support. This was unexpected given prior twin studies highlighting the heritability of perceived social support (Coventry et al., 2021) and suggesting that genetic factors account for 40%–55% of its variance in late adolescence, with shared genetic overlap with mental health outcomes (Matthews et al., 2016; Wang et al., 2017). However, given the paucity of twin studies that have examined the aetiology of social support in early life (Wang et al., 2017), it remains unclear whether these estimates can be generalized to preadolescence. The current study helps address this gap by revealing indirect associations between the polygenic scores for internalizing and externalizing behaviours and perceived social support through corresponding behavioural manifestations at age 11. Taken together, these findings suggest that higher genetic predispositions for internalizing and externalizing problems may contribute to greater mental health difficulties in preadolescence, which, in turn, are associated with reduced perceptions of social support. These results warrant replication, particularly during adolescence, to further elucidate the genetic and developmental underpinnings of perceived social support.

Second, this study documented that children with greater genetic predispositions towards internalizing and externalizing behaviours were more likely to experience childhood adversity, revealing a process of gene‐environment correlation. Conversely, the internalizing and externalizing polygenic scores were not predictive of exposure to perinatal adversity, nor was the polygenic score for educational attainment protective against ACEs. Some of these findings align with a growing body of evidence showing that ACEs do not occur randomly across the population and are associated with genetic vulnerability to neurodevelopmental and mental health disorders, including attention‐deficit/hyperactivity disorder, schizophrenia and depression (Baldwin et al., 2022; Bolhuis et al., 2022; Sallis et al., 2021; Schoeler et al., 2019; Zwicker et al., 2020). Our study extends these findings by demonstrating that gene‐environment correlations may be specific to psychosocial ACEs that unfold during childhood. Our results also suggest that this timing effect may dissipate when associations are examined by adversity subtypes. Indeed, our analyses showed that the polygenic scores were related to specific subtypes of adversity experienced either in the perinatal period or in childhood. Specifically, higher genetic predispositions for educational attainment were related to higher incidence of preterm birth and less exposure to parental externalizing problems during childhood. In addition, higher genetic predispositions for internalizing behaviours were associated with greater exposure to maternal physical or psychological problems in the first month after birth. Significant associations were also documented between genetic predispositions for internalizing and externalizing behaviours and each subtype of childhood adversity, with the exception of out‐of‐home placement. Collectively, these findings underline the complex nature of the associations unfolding between adolescents' genetic predispositions and both the timing of exposure and subtypes of ACEs. Considering that the ACEs assessed in this study occurred within the family environment, a potential underlying mechanism for these associations could be passive gene‐environment correlations. Specifically, parents may create a home environment that is influenced by their own genotypes, leading to an association between the genetic predispositions they transmit to their offspring and the environment in which their children are raised (Bolhuis et al., 2022; Sallis et al., 2021). Further research is needed to test this hypothesis and identify actionable family pathways driving these correlations. Such pathways could be targeted by interventions to mitigate the impact of genetic predispositions for internalizing and externalizing behaviours on later adversity, reducing risks and fostering resilience in youth.

Third, we found a complex pattern of association between ACEs and perceived support from parents in preadolescence, although their magnitudes were small. Unexpectedly, exposure to perinatal adversity, mainly preterm birth and maternal physical or psychological problems 1 month after birth, was associated with greater support from mothers. This contrasts with evidence from several studies reporting a negative association between ACEs and perceived social support (McCoy et al., 2020; Turner et al., 2017; Yearwood et al., 2019). Preterm birth is associated with perinatal complications (Luciana, 2003) and increased risk of later developmental and behavioural problems (O’Nions et al., 2021). These risks may lead mothers to perceive their preterm adolescents as particularly vulnerable, promoting more nurturing behaviours (Toscano et al., 2022; Whittingham et al., 2014), which may enhance youths' perception of maternal support.

In contrast, different patterns emerged for childhood psychosocial adversity. A parental history of internalizing problems was correlated with lower perceived support from mothers, whereas low family income, parental divorce and parental history of externalizing behaviours were associated with lower perceived support from fathers. These findings converge with prior work (McCoy et al., 2020; Turner et al., 2017; Yearwood et al., 2019), but extend it by highlighting distinct associations with maternal and paternal support. Of note, we measured perceived, not enacted, support, which are only moderately correlated. Thus, lower perceived support may not reflect less actual support. However, as perceived support is more strongly associated with mental health and well‐being (Rueger et al., 2016), promoting a strong perception of parental support is essential for positive development.

Although smaller in magnitude than previous studies (McCrory et al., 2022; Turner et al., 2017), these associations carry practical implications. The opposing associations of prenatal and childhood ACES with parental support suggest that not all adversity is related to lower perceived support. Intervention efforts may benefit from considering potential impacts of specific adversity types, developmental timing and support sources to better promote resilience. Our results also underscore the value of programs that foster strong father‐child relationships post‐divorce (Sandler et al., 2018) and point to the potential benefits of addressing fathers' externalizing behaviours and financial strain. Further research is needed to clarify the role of perceived social support in youth resilience, which may ultimately inform tailored intervention efforts.

The present study has several strengths, including its extensive sample size, thorough assessment of ACEs spanning pre‐postnatal and childhood periods, and examination of perceived social support from multiple sources (mothers, fathers, and friends). Additionally, the incorporation of polygenic scores encompassing both developmentally‐enhancing (educational attainment) and risk‐promoting (internalizing and externalizing behaviours) genetic predispositions enhances the depth of our analysis, allowing for a nuanced exploration of associations between the timing and subtypes of adversity with genetic liabilities and perceived social support.

That said, this study is not without limitations. Firstly, most ACE indicators were assessed retrospectively via parent interviews at age 11, with 95% of respondents being mothers and 5% fathers, potentially introducing recall bias. Future research should validate our findings using prospective ACE assessments. Secondly, our focus on ACEs within the home environment limits our ability to assess how genetic predispositions interact with broader environmental factors in affecting perceived social support. Moreover, the ACEs factors reflected distinct experiences, making it difficult to disentangle the impact of timing from the nature of adversity. Expanding the scope to include diverse ACEs and positive environmental influences measured repeatedly could provide further insights. Lastly, the study's homogeneous sample of white Dutch adolescents restricts the generalizability of our findings to more culturally and ethnically diverse populations, highlighting the need for research in varied demographic contexts.

## CONCLUSION

In conclusion, this study demonstrated that adolescents predisposed to internalizing and externalizing behaviours are more susceptible to experience childhood adversity. Such experiences were associated with lower perceived paternal support in preadolescence, whereas perinatal adversity correlated with higher perceived maternal support. Further analyses revealed that higher genetic predispositions for internalizing and externalizing problems were indirectly related to lower perceived social support. Together, these findings highlight the complexity of the associations unfolding between genetic predispositions, ACEs and perceived social support, underscoring the importance of considering both the timing and subtypes of adversity, as well as distinct sources of support, in future research.

## AUTHOR CONTRIBUTIONS

**Christina Y. Cantave**: Conceptualization; formal analysis; visualization; writing—original draft; writing—review and editing. **Marthe de Roo**: Formal analysis; writing—review and editing. **Charlotte Vrijen**: Formal analysis; writing—review and editing. **Tina Kretschmer**: Conceptualization; funding acquisition; supervision; writing—review and editing.

## CONFLICT OF INTEREST STATEMENT

The authors declare no conflicts of interest.

## ETHICAL CONSIDERATIONS

Inform consent/assent from both parents and children has been appropriately obtained and ethical approval granted by the Dutch national ethics committee (Population cohort: P00.0246C [wave 1: 2000] and P03.105C [wave 3: 2006]; high‐risk cohort: P03.1700C [wave 1: 2004] and NL21154.042.07 [wave 3: 2009]).

## Supporting information

Supporting Information S1

## Data Availability

The data that support the findings of this study are available from TRAILS and is subject to the European Union's General Data Protection Regulation. Data can be requested from TRAILS, at [https://www.trails.nl/en/researchers/working‐with‐trails] and can be requested.
