# Supplementary material for: Investigating the impact of early adversity on perceived support from parents and friends in preadolescence: Do genetic predispositions and timing of exposure matter?
Source: JCPP Adv. 2026 Jan 20:e70090. Online ahead of print. doi: 10.1002/jcv2.70090 (PMC13338975; doi:10.1002/jcv2.70090)
Supplement: Supplementary file 1 — Supporting Information S1 [file JCV2-9999-e70090-s001.docx]

**Investigating the impact of early adversity on perceived support from parents and friends in preadolescence: Do genetic predispositions and timing of exposure matter?**

**Supporting Information**

Table of Contents

Appendix S1. [Estimation of pre-postnatal and childhood adversity factors 2](#_Toc210986243)

Appendix S2. [Genotyping Procedure 4](#_Toc210986244)

Appendix S3. [Explained variance by the polygenic scores (PGSs) 4](#_Toc210986245)

[Table S1. Explained variance in phenotypes by the polygenic scores 5](#_Toc210986246)

[Table S2. Associations between perinatal adversity indicators, polygenic scores and social support 7](#_Toc210986247)

[Table S3. Associations between childhood adversity indicators, polygenic scores and social support 8](#_Toc210986248)

[Table S4. Indirect effects of the polygenic scores on perceived support from parents and friends 9](#_Toc210986249)

# Appendix S1. Estimation of pre-postnatal and childhood adversity factors

Pre-and postnatal adversity was derived using data collected at T1, encompassing birth weight, gestational age, mother’s hospitalization during pregnancy, child’s hospitalization for 10 days or more immediately after birth, maternal physical and psychological problems during the first month after delivery. Preterm birth (≤ 33 weeks) and low birth weight (≤2500 g [≈5.51 lb]) were derived from information on birth weight and gestational age. The remaining indicators were assessed in at-home interviews with parents when the child was age 11, conducted by well-trained interviewers using the following questions: “*Was [the mother] hospitalized during the pregnancy?*”, “*Did [name of child] have to stay or be hospitalized after birth*”; “*How many days did [name of child] have to stay or be hospitalized after birth?*”; “*Did [the mother] have any physical or psychological problems in the first month after birth?*”. Of note, more than a third (32%) of children were hospitalized after birth. To ensure that we captured those who were exposed to significant adversity, a more stringent criterion was applied, classifying children hospitalized for 10 days or more after birth (equivalent to the 75th percentile of the number of days children were hospitalized) as being exposed to adverse experiences. Each indicator was dummy coded, with 0 designating “*little to no exposure*” and 1 denoting “*exposure*.”

The childhood adversity score was based on five adverse experiences that occurred when the child was aged between 0 and 11. These events included low family income (<€1,135 per month at T1), parental divorce (“*Did [name of child] experience parental divorce or separation?*”), out-of-home placement (“*Has [name of child] ever been away from home for 3 months or more?*”) and parental history of mental health problems. The latter was assessed through the TRAILS Family History Interview (FHI), administered during the parental interview. Five dimensions of lifetime psychopathology were evaluated—depression, anxiety, substance dependence, persistent antisocial behaviour, and psychosis. Each dimension was introduced with a vignette describing DSM-IV characteristics, followed by questions on lifetime occurrence, professional treatment, and medication use. Parents were categorized as 0= “*never had an episode*,” 1= “*yes*,” 2= “*yes with treatment/medication*”, or 2= “*yes with police involvement for antisocial behaviour*”. Prevalence rates in mothers and fathers, respectively, were, for depression: 27% and 15%; for anxiety: 16% and 6%; for substance dependence: 3% and 7%; and for antisocial behaviour: 3% and 7%. From these, we created indices of parental history for two broad domains of mental disorders, namely internalizing (anxiety and depression) and externalizing (substance dependence and antisocial behaviour) disorders. Both domains were a count of the number of lifetime disorders within each domain reported by biological parents (for more information, see Ormel et al., 2005).

The prevalence of adverse childhood experiences (ACEs) in our sample was as follows in the pre-postnatal period: 58.4% for no ACE, 28.4% for one ACE; 8.5% for two ACEs, 3% for three ACEs and 1.8% for 4 or more ACEs. In childhood, the prevalence of ACEs was 44.8% for no ACE, 31.8% for one ACE; 13.6% for two ACEs, 7.3% for three ACES and 2.5% for 4 or more ACEs.

Consistent with best practice (Wright & Schwartz, 2021), all items were included in confirmatory factor analyses (CFA) to derive robust and cohesive latent factors of adversity during the pre-and postnatal and childhood periods. A CFA approach was preferred over a cumulative risk index for several reasons: First, by evaluating the loading of each indicator on the overall factor, CFA verifies that each indicator makes a meaningful contribution to the estimation of the underlying adversity factors. Second, latent variables have been shown to possess greater predictive power for various outcomes compared to cumulative scores (Hall et al., 2010; Wright & Schwartz, 2021). The specific perinatal adverse indicators were all positively related, which was also the case for the childhood adversity indicators (see Tables S2 and S3 below).

# Appendix S2. Genotyping Procedure

DNA was extracted from blood samples (*n*=1565) or, in a small proportion of samples, buccal swabs (Cytobrush; *n*=360) using a manual salting out procedure (Miller et al., 1988). Genotyping was performed on the Golden Gate Illumina BeadStation 500 and the Infinium™ HumanCytoSNP-12 v2.1 BeadChip platforms (Illumina Inc., San Diego, CA), according to the manufacturers’ protocols. These datasets were merged and checked for genotype concordance. DNA samples that were too heterogeneous, were duplicated or related, or were from non-European descent (as determined by principal component analysis of our samples combined with all 1000G samples) were removed. In addition, genetic variants with >5% missing data, minor allele frequency <1%, or deviated significantly from Hardy-Weinberg equilibrium (*p* <10-6) were excluded. One SNP showed >5% mismatches and was excluded from the Golden Gate dataset after checking the minor allele frequency with HapMap. Genotypes were next imputed using the Haplotype Reference Consortium’s global reference panel on the Michigan Imputation server (Das et al., 2016; McCarthy et al., 2016). We excluded one member of all sibling pairs (*n*=32), retaining the sibling for whom most data were available. If this was the same for both siblings, we randomly selected one sibling. This resulted in a total sample of *n*=1678 with valid genetic data.

# Appendix S3. Explained variance by the polygenic scores (PGSs)

Following the estimation of PGSs, we conducted additional analyses to evaluate the variance in educational attainment, internalizing and externalizing behaviours explained by the PGSs. As shown in Table S1, each PGS significantly predicted variance in its respective phenotype, although the effect sizes were small. Moreover, when we compared our results to those of a previous study using this sample (Vrijen et al., 2023), we noted that the internalizing and externalizing PGSs explained slightly more variance in internalizing and externalizing behaviours self-reported at age 16. This finding aligns with the results shown in Table S1, indicating higher explained variance for educational attainment measured at ages 14 and 16. Taken together, these results seem to suggest that PGSs estimated using genome-wide association studies on adult phenotypes may not fully capture the genetic variants associated with phenotypes measured in early adolescence. This emphasizes the need for well-powered GWAS focused on adolescent populations to better understand the genetic architecture of these behaviours during this critical developmental period (Jami et al., 2022).

# Table S1. Explained variance in phenotypes by the polygenic scores

| Phenotypes | PGSs | *R*^2^ | *p* |
| --- | --- | --- | --- |
| Internalizing behaviours (age 11) | Internalizing PGS | 0.006 | 0.002 |
| Externalizing behaviours (age 11) | Externalizing PGS | 0.007 | $<$0.001 |
| Progress during primary school (age 11) | Educational attainment PGS | 0.003 | 0.033 |
| Verbal and spatial intelligence (age 11) | Educational attainment PGS | 0.006 | 0.001 |
| Educational level (age 14) | Educational attainment PGS | 0.011 | $<$0.001 |
| Educational level (age 16) | Educational attainment PGS | 0.016 | $<$0.001 |

Notes. Internalizing behaviours were self-reported using the internalizing subscales of the Youth Self Report (YSR) questionnaire (31 items α=0.87; Achenbach & Rescorla, 2001). Externalizing behaviours were self-reported using the externalizing subscales of the YSR (32 items, α=0.85). Verbal and spatial intelligence was measured using the Revised Wechsler Intelligence Scale for Children (WISC-R; Silverstein, 1975). Progress during primary school was measured as follows: -3 = Special education, -2 = Repeated grade twice, -1 = Repeated grade once, 0 = Regular progress, 1 = Skipped a class. Educational level at ages 14 and 16 was measured as follows: 1 = Lower vocational track, 2 = Intermediate vocational track, 3 = Higher vocational track, 4 = Academic track (for more information, see Schmengler et al., 2023).

# Table S2. Associations between perinatal adversity indicators, polygenic scores and social support

|  | 1 | 2 | 3 | 4 | 5 | 6 | 7 | 8 | 9 | 10 | 11 |
| --- | --- | --- | --- | --- | --- | --- | --- | --- | --- | --- | --- |
| 1. Preterm birth | ⎯ | 61.35*** | 3.88*** | 44.35*** | 2.59*** | -.02 | .01 | .05* | .04* | .01 | .03 |
| 1. Low birth weight |  | ⎯ | 2.58*** | 11.40*** | 1.48** | .03 | .001 | .04 | .01 | -.03 | .02 |
| 1. Mother hospitalization during pregnancy |  |  | ⎯ | 4.10*** | 2.27*** | .01 | -.01 | -.01 | .03 | -.003 | -.02 |
| 1. Child hospitalization for 10 days or more after birth |  |  |  | ⎯ | 3.19*** | .01 | .03 | -.02 | .03 | .03 | -.001 |
| 1. Maternal physical or psychological in the first month after birth |  |  |  |  | ⎯ | .06* | .03 | .01 | .04* | .04* | .02 |
| 1. Internalizing PGS |  |  |  |  |  | ⎯ | .25*** | -.01 | .004 | .01 | -.02 |
| 1. Externalizing PGS |  |  |  |  |  |  | ⎯ | .01 | -.01 | -.03 | .02 |
| 1. Educational attainment PGS |  |  |  |  |  |  |  | ⎯ | .02 | -.01 | -.01 |
| 1. PS from mothers |  |  |  |  |  |  |  |  | ⎯ | .59*** | .37*** |
| 1. PS from fathers |  |  |  |  |  |  |  |  |  | ⎯ | .37*** |
| 1. PS from friends |  |  |  |  |  |  |  |  |  |  | ⎯ |

Notes. PGS = polygenic score; PS= perceived support. All adversity indicators are coded as follows: 0 for little to no exposure and 1 for exposure. Odds ratios are provided for associations involving adversity indicators, while standardized beta coefficients are presented for all other associations.*** *p* $\leq$ 0.001, ** *p* $\leq$ 0.01, * *p* $\leq$ 0.05.

# Table S3. Associations between childhood adversity indicators, polygenic scores and social support

|  | 1 | 2 | 3 | 4 | 5 | 6 | 7 | 8 | 9 | 10 | 11 |
| --- | --- | --- | --- | --- | --- | --- | --- | --- | --- | --- | --- |
| 1. Low family income | ⎯ | 6.64*** | 3.18*** | 1.50*** | 2.78*** | .11*** | .14*** | .03 | .01 | -.05* | -.004 |
| 1. Parental divorce |  | ⎯ | 6.60*** | 2.51*** | 7.15*** | .09*** | .14*** | -.04 | .02 | -.06** | .03 |
| 1. Out-of-home placement |  |  | ⎯ | 2.49** | 5.16*** | .03 | .02 | -.03 | -.02 | -.02 | .03 |
| 1. Parental history of internalizing problems |  |  |  | ⎯ | 4.23*** | .07** | .07** | -.01 | -.04* | -.02 | .001 |
| 1. Parental history of externalizing problems |  |  |  |  | ⎯ | .10*** | .12*** | -.06* | .01 | -.06** | -.01 |
| 1. Internalizing PGS |  |  |  |  |  | ⎯ | .25*** | -.01 | .004 | .01 | -.02 |
| 1. Externalizing PGS |  |  |  |  |  |  | ⎯ | .01 | -.01 | -.03 | .02 |
| 1. Educational attainment PGS |  |  |  |  |  |  |  | ⎯ | .02 | -.01 | -.01 |
| 1. PS from mothers |  |  |  |  |  |  |  |  | ⎯ | .59*** | .37*** |
| 1. PS from fathers |  |  |  |  |  |  |  |  |  | ⎯ | .37*** |
| 1. PS from friends |  |  |  |  |  |  |  |  |  |  | ⎯ |

Notes. PGS = polygenic score, PS= perceived support. All adversity indicators are coded as follows: 0 for little to no exposure and 1 for exposure. Odds ratios are provided for associations involving adversity indicators, while standardized beta coefficients are presented for all other associations.*** *p* $\leq$ 0.001, ** *p* $\leq$ 0.01, * *p* $\leq$ 0.05.

# Table S4. Indirect effects of the polygenic scores on perceived support from parents and friends

| Predictor | Mediator | Outcome (Perceived support) | Total effect (*b*; *p*) | Indirect effect (*b*; *p*) | Direct effect (*b*; *p*) |
| --- | --- | --- | --- | --- | --- |
| Internalizing PGS | Internalizing behaviours (age 11) | Mother | -0.004; 0.858 | **-0.012; 0.002** | 0.008; 0.741 |
|  |  | Father | 0.005; 0.854 | **-0.013; 0.002** | 0.017; 0.495 |
|  |  | Friends | -0.037; 0.134 | **-0.014; 0.001** | -0.023; 0.352 |
| Externalizing PGS | Externalizing behaviours (age 11) | Mother | -0.022; 0.753 | **-0.022; 0.001** | 0.014; 0.556 |
|  |  | Father | -0.030, 0.241 | **-0.021; 0.001** | -0.010; 0.697 |
|  |  | Friends | 0.015; 0.537 | **-0.013; 0.003** | 0.028; 0.245 |
| Educational attainment PGS | Progress during primary school (age 11) | Mother | 0.023; 0.321 | 0.004; 0.214 | 0.019; 0.415 |
|  |  | Father | -0.011; 0.668 | 0.001; 0.717 | -0.011; 0.645 |
|  |  | Friends | -0.027; 0.297 | <0.001; 0.969 | -0.027; 0.296 |

Notes. Models are adjusted for sex, age, and the first 20 principal components. Significant associations are in bold. Internalizing behaviours were self-reported using the internalizing subscales of the Youth Self Report (YSR) questionnaire (31 items α=0.87; Achenbach & Rescorla, 2001). Externalizing behaviours were self-reported using the externalizing subscales of the YSR (32 items, α=0.85). Progress during primary school was measured as follows: -3 = Special education, -2 = Repeated grade twice, -1 = Repeated grade once, 0 = Regular progress, 1 = Skipped a class.

**References**

Achenbach, T., & Rescorla, L. A. (2001). *Manual for the ASEBA school-age forms and profiles*. Burlington, VT: University of Vermont, Research center for children, youth and families. www.aseba.org

Das, S., Forer, L., Schönherr, S., Sidore, C., Locke, A. E., Kwong, A., Vrieze, S. I., Chew, E. Y., Levy, S., McGue, M., Schlessinger, D., Stambolian, D., Loh, P.-R., Iacono, W. G., Swaroop, A., Scott, L. J., Cucca, F., Kronenberg, F., Boehnke, M., … Fuchsberger, C. (2016). Next-generation genotype imputation service and methods. *Nature Genetics*, *48*(10), Article 10. https://doi.org/10.1038/ng.3656

Hall, J. E., Sammons, P., Sylva, K., Melhuish, E., Taggart, B., Siraj-Blatchford, I., & Smees, R. (2010). Measuring the combined risk to young children’s cognitive development: An alternative to cumulative indices. *British Journal of Developmental Psychology*, *28*(2), 219–238. https://doi.org/10.1348/026151008X399925

Jami, E. S., Hammerschlag, A. R., Ip, H. F., Allegrini, A. G., Benyamin, B., Border, R., Diemer, E. W., Jiang, C., Karhunen, V., Lu, Y., Lu, Q., Mallard, T. T., Mishra, P. P., Nolte, I. M., Palviainen, T., Peterson, R. E., Sallis, H. M., Shabalin, A. A., Tate, A. E., … Middeldorp, C. M. (2022). Genome-wide Association Meta-analysis of Childhood and Adolescent Internalizing Symptoms. *Journal of the American Academy of Child & Adolescent Psychiatry*, *61*(7), 934–945. https://doi.org/10.1016/j.jaac.2021.11.035

McCarthy, S., Das, S., Kretzschmar, W., Delaneau, O., Wood, A. R., Teumer, A., Kang, H. M., Fuchsberger, C., Danecek, P., Sharp, K., Luo, Y., Sidore, C., Kwong, A., Timpson, N., Koskinen, S., Vrieze, S., Scott, L. J., Zhang, H., Mahajan, A., … the Haplotype Reference Consortium. (2016). A reference panel of 64,976 haplotypes for genotype imputation. *Nature Genetics*, *48*(10), Article 10. https://doi.org/10.1038/ng.3643

Miller, S. A., Dykes, D. D., & Polesky, H. F. (1988). A simple salting out procedure for extracting DNA from human nucleated cells. *Nucleic Acids Research*, *16*(3), 1215.

Ormel, J., Oldehinkel, A. J., Ferdinand, R. F., Hartman, C. A., Winter, A. F. D., Veenstra, R., Vollebergh, W., Minderaa, R. B., Buitelaar, J. K., & Verhulst, F. C. (2005). Internalizing and externalizing problems in adolescence: General and dimension-specific effects of familial loadings and preadolescent temperament traits. *Psychological Medicine*, *35*(12), 1825–1835. https://doi.org/10.1017/S0033291705005829

Silverstein, A. B. (1975). Validity of WISC-R short forms. *Journal of Clinical Psychology*, *31*(4), 696–697. https://doi.org/10.1002/1097-4679(197510)31:4<696::AID-JCLP2270310429>3.0.CO;2-M

Vrijen, C., Nolte, I. M., Oldehinkel, A. J., Veenstra, R., & Kretschmer, T. (2023). Genetic confounding in bullying research: Causal claims revisited. *Development and Psychopathology*, 1–12. https://doi.org/10.1017/S0954579423000445

Wright, E. M., & Schwartz, J. A. (2021). The influence of adverse childhood experiences on internalizing and externalizing problems in early adulthood: Evidence of a gene × environment × sex interaction. *Child Abuse & Neglect*, *114*. https://doi.org/10.1016/j.chiabu.2021.104962
